# Management of Pregnancy with Cervical Shortening: Real-Life Clinical Challenges

**DOI:** 10.3390/medicina59040653

**Published:** 2023-03-26

**Authors:** Anna Kornete, Ludmila Volozonoka, Maksims Zolovs, Adele Rota, Inga Kempa, Linda Gailite, Dace Rezeberga, Anna Miskova

**Affiliations:** 1 Department of Obstetrics and Gynaecology, Riga Stradins University, LV-1007 Riga, Latvia; 2 Riga Maternity Hospital, LV-1013 Riga, Latvia; 3 Scientific Laboratory of Molecular Genetics, Riga Stradins University, LV-1007 Riga, Latvia; 4 Department of Statistics, Riga Stradins University, LV-1007 Riga, Latvia; 5 Institute of Life Sciences and Technologies, Daugavpils University, LV-5401 Daugavpils, Latvia

**Keywords:** preterm birth, short cervix, cervical insufficiency, progesterone, intra-amniotic infection, uterine cervix, pessary, cerclage

## Abstract

*Background and Objectives:* Preterm birth is the leading cause of neonatal mortality worldwide and may be responsible for lifelong morbidities in the survivors. Cervical shortening is one of the common pathways to preterm birth associated with its own diagnostic and management challenges. The preventive modalities that have been tested include progesterone supplementation and cervical cerclage and pessaries. The study aimed to assess the management strategies and outcomes in a group of patients with a short cervix during pregnancy or cervical insufficiency. *Materials and Methods:* Seventy patients from the Riga Maternity Hospital in Riga, Latvia, were included in the prospective longitudinal cohort study between 2017 and 2021. Patients were treated with progesterone, cerclage, and/or pessaries. The signs of intra-amniotic infection/inflammation were assessed, and antibacterial therapy was given when the signs were positive. *Results:* The rates of PTB were 43.6% (*n* = 17), 45.5% (*n* = 5), 61.1% (*n* = 11), and 50.0% (*n* = 1) in progesterone only, cerclage, pessary, and cerclage plus pesssary groups, respectively. The progesterone therapy was associated with a reduced preterm birth risk (x2(1) = 6.937, *p* = 0.008)), whereas positive signs of intra-amniotic infection/inflammation significantly predicted the risk of preterm birth (*p* = 0.005, OR = 3.82, 95% [CI 1.31–11.11]). *Conclusions:* A short cervix and bulging membranes, both indicators of intra-amniotic infection/inflammation, are the key risk factors in preterm birth risk predictions. Progesterone supplementation should remain at the forefront of preterm birth prevention. Among patients with a short cervix and especially complex anamnesis, the preterm rates remain high. The successful management of patients with cervical shortening lies between the consensus-based approach for screening, follow-up, and treatment on the one side and personalising medical therapy on the other.

## 1. Introduction

The World Health Organisation defines preterm birth (PTB) as childbirth before 37 weeks of gestation. The incidence of PTB is estimated to be 5–18% worldwide and 5.2–10.4% in Europe [1]. PTB remains the leading cause of neonatal mortality and short- and long-term morbidity, as well as a significant social and economic burden [2]. Cervical shortening is one of the common pathways to PTB. Spontaneous PTB and cervical shortening (effacement) are both considered multifactorial syndromes [3,4].

### 1.1. A Short Cervix and Cervical Insufficiency

A short cervix is defined as a cervical length (CL) of ≤25 mm in a transvaginal sonographic examination (TVS) before 24 weeks of gestation [5,6], although some authors consider cervical shortening even before 32 weeks of gestation [7,8,9]. Causes of cervical shortening include intra-amniotic (IA) infection or sterile inflammation (defined as the absence of demonstrable microorganisms in the amniotic fluid), decidual haemorrhage, uterine overdistension, disruption of maternal–foetal tolerance, and others. These clinical situations can lead to preterm cervical shortening and often a single (non-recurrent) second-trimester pregnancy loss or PTB [10,11].

In contrast, the probable cause of a recurrent second-trimester pregnancy loss or PTB is a short cervix due to the structural changes leading to the condition of cervical insufficiency (CI). The definition of CI by the American College of Obstetricians and Gynaecologists is ‘the inability of the uterine cervix to retain pregnancy in the second trimester in the absence of clinical contractions, labour, or both’ [12]. Structural cervical changes may be acquired (i.e., secondary to a prior cervical or uterine surgery, e.g., a dilation and curettage, a hysteroscopy, a loop electrosurgical excision procedure, or conization) or congenital (e.g., arcuate uterus, septate uterus) [11]. Furthermore, a group of genetic disorders affecting the connective tissues (e.g., the Ehlers–Danlos syndrome, the Marfan syndrome) are known risk factors for cervical shortening during pregnancy [11,13]. In the previous study, our group revealed 12 genes primarily related to CI, with the majority known to cause collagenopathies, thus proposing the idea of CI as a subtle form of collagenopathy [14].

### 1.2. The Current Management Strategies of a Short Cervix and Cervical Insufficiency

Patients with a short cervix or CI can be treated with vaginal progesterone, which reduces the rate of spontaneous PTB and neonatal morbidity [15,16]. According to the 2022 guidelines of The International Society of Ultrasound in Obstetrics and Gynaecology (ISUOG) for asymptomatic women with singleton pregnancy without prior spontaneous PTB and with CL ≤ 25 mm in TVS before 24 weeks, the administration of natural vaginal progesterone is recommended from the time of detection of the short cervix until 36 weeks of gestation [17]. In women with a singleton gestation and prior spontaneous PTB, treatment with vaginal progesterone every night from 16 to 36 weeks, or surveillance and treatment in those with CL ≤ 25 mm, should be considered, although there is still conflicting evidence [17].

Cervical cerclage can be considered in women with a singleton pregnancy without prior spontaneous PTB, whose cervix shortens to <10 mm despite being on progesterone [17,18,19]. In women with a singleton gestation and prior spontaneous PTB who are on progesterone and have a TVS CL ≤ 25 mm on CL screening before 24 weeks, cerclage may be recommended [17]. In turn, the placement of a cervical pessary has been considered controversial [20,21]. Current evidence does not support the use of cervical pessaries to prevent PTB in asymptomatic patients with or without prior spontaneous PTB with CL < 25 mm outside the research protocols [17].

### 1.3. The Aim of the Study

As noted, the evaluation strategy in patients with cervical shortening and CI is challenged by the heterogenous aetiology of the condition. Furthermore, the optimal management of patients with cervical shortening, regarding the decision of when to start progesterone and when or whether to place cerclage or a pessary, is clinically burdensome. The purpose of this study was to identify the key risk factors in PTB risk prediction as well as to assess the existing management strategies and pregnancy outcomes among patients with cervical shortening during pregnancy in a single centre in Latvia.

## 2. Materials and Methods

### 2.1. Research Ethics

The study was conducted according to the ethical principles of the Declaration of Helsinki. The study protocol was approved by the Central Medical Ethics Committee of Latvia (No. 2/18–03–21). Written informed consent was obtained from all participants before enrolment in the study. The characteristics of the participants, including details of maternal age, race, height, weight, history of cervical surgery and obstetric history, and data on the pregnancy outcome, were obtained from medical records, and the questionnaire was completed by the patient.

### 2.2. Patient Inclusion and Exclusion Criteria

Between 2017 and 2021, seventy patients from the Riga Maternity Hospital in Riga, Latvia, were included in the prospective longitudinal cohort study. Women attending the Riga Maternity Hospital with a history of second-trimester pregnancy loss or extreme PTB (<28 weeks) or the ones undergoing routine antenatal care were examined for a short cervix or CI and recruited at the point of requiring treatment for cervical shortening. The inclusion criteria were a singleton pregnancy and a short cervix or a CI.

The diagnosis of cervical shortening was defined as one of the following:

1. An obstetric history-based diagnosis—CI in the patient with a classic history of ≥2 consecutive prior second-trimester pregnancy losses or PTB before 28 weeks associated with no or minimal symptoms of pelvic pressure, Braxton Hicks-like contractions, premenstrual-like cramping and/or backache, or change in vaginal discharge;
2. A ultrasound-based diagnosis—CL ≤ 25 mm before 28 weeks in the current pregnancy;
3. A physical examination-based diagnosis—a dilated and effaced cervix in a physical examination, and no contractions or weak irregular contractions that appeared inadequate to explain the cervical changes with or without prolapsed or ruptured membranes before 28 weeks.

We excluded patients younger than 18 years of age or in active labour. 

Spontaneous preterm labour was defined by the presence of regular uterine contractions (≥4 every 20 min) associated with the cervical change before 37 weeks.

The standardisation of CL examination in TVS was achieved using the technique described by the ISUOG [17]. The patient was placed in the dorsal lithotomy position with an empty bladder. The TVS probe was inserted in the anterior fornix of the vagina without pressure on the cervix. The sagittal view of the cervix was obtained, and callipers were used to measure the linear distance between the triangular area of echo density at the external os and the V-shaped notch at the internal os. Each examination was performed for a period of 2–3 min by an experienced clinician.

### 2.3. Assessment of Intraamniotic Infection and Inflammation

The IA infection/inflammation group was defined when the cervix was dilated by ≥2 cm on a digital or a speculum examination, OR the TVS findings were consistent with IA inflammation (debris in the amniotic fluid (sludge)), OR the membranes were bulging (membranes were visible and exposed at or beyond the external os with cervix dilatation of <2 cm). Patients were divided into two groups according to the clinical signs of IA infection/inflammation (the IA infection/inflammation group and the non-infection/inflammation group).

### 2.4. The Treatment Principles

In patients recruited for progesterone treatment, 200 mg vaginal progesterone was prescribed daily at the time of the diagnosis of cervical shortening. Progesterone supplementation was continued until 37 weeks. For the women assigned to a cervical cerclage, the transvaginal McDonald cerclage with 5 mm Mersilene tape was performed by an experienced clinician. For the women assigned to a cervical pessary, the Arabin pessary was used. The cerclage and pessary were removed in the event of a confirmed preterm premature rupture of membranes (PPROM) and/or regular contractions. Otherwise, both the cerclage and pessary were removed in an outpatient procedure at 37 weeks. In patients with signs of IA infection/inflammation, an antibacterial therapy was prescribed.

### 2.5. Statistical Data Analysis

To compare the IA infection/inflammation group with the non-infection/inflammation group, the Mann–Whitney U test was conducted to determine whether there was a significant difference between the two independent groups. The chi-square test of independence was conducted to determine whether there was an association between the two variables. Binomial logistic regression was conducted to determine how the numerous independent variables (progesterone, cerclage, pessary groups) are related to a dichotomous dependent variable. Akaike’s Information Criterion was used as a comparative measure of a model fit. The *p*-values for all hypothesis tests were two-sided, and statistical significance was set at *p* < 0.05. Statistical analysis was performed using Jamovi software.

## 3. Results

### 3.1. The Baseline Characteristics of the Study Population

A total of 70 patients with cervical shortening were included in the study. The baseline characteristics of the study population are shown in Table 1.

### 3.2. The Comparison of the Intra-Amniotic Infection/Inflammation Group and the Non-Infection/Inflammation Group

The signs of IA infection/inflammation were observed in 27 (38.6%) patients: the TVS findings were consistent with IA inflammation (sludge) in 12 (44.4%), cervical dilatation of ≥2 cm in 21 (77.8%), and bulging membranes in 14 (51.9%) patients. In 15 (55.6%) cases, more than one sign of IA infection/inflammation was observed. The presence of the signs of IA infection/inflammation significantly predicted the risk of PTB (*p* = 0.014, OR = 3.82, 95% [CI 1.31–11.11]). Specifically, the chi-squared test showed that the presence of cervical dilatation and bulging membranes was associated with the risk of PTB (*p* = 0.01, OR 2.9 95%, CI [1.2–6.9], and *p* = 0.031, RR 3.3 95%, TI [1.0–10.7], respectively). In the IA infection/inflammation group, PTB before 27 + 6 weeks occurred in 37.1%, PTB between 28 + 0–31 + 6 weeks and between 32 + 0–36 + 6 weeks occurred in 11.1 and 11.1% (x^2^ (2 = 6.125, *p* = 0.048)), respectively (Table 2).

Perinatal mortality was higher in the IA infection/inflammation group (*p* = 0.048). PTB or second-trimester pregnancy loss, a 1 min Apgar score of <7, and perinatal mortality were more common in the IA infection/inflammation group (*p* = 0.009). Maternal septic complications were observed in six (8.6%) patients from the study population. No statistically significant correlation between IA infection/inflammation and maternal septic complication was found (*p* > 0.05). Chorioamnionitis was diagnosed in four patients, with endometritis and sepsis in one case.

In total, 46 (65.7%) patients from the study population received antibacterial therapy. Twenty-seven (58.7%) of these patients were from the IA infection/inflammation group. There were 54.3% of PTBs in the group who had received antibacterial therapy and 57.0% in the group without antibacterial therapy; thus, antibacterial therapy was not associated with a reduced risk of PTB.

### 3.3. Progesterone, Cerclage, and Pessary

All patients received treatment with progesterone, including patients in the cerclage group and the pessary group. A cerclage was placed in 13 (18.6%) patients. In four patients, a cerclage was placed due to an obstetric history-based diagnosis. Furthermore, in one of these patients, an elective transabdominal cerclage was performed pre-pregnancy due to the previous failure of the transvaginal cerclage. A cerclage, due to ultrasound-based diagnosis, was performed in nine cases. The mean timing of a cerclage placement was 17 weeks when an average CL was 15 mm. Eight (61.5%) patients with an ultrasound-based diagnosis had experienced one or more PTB or second-trimester losses in the anamnesis. All the patients in the cerclage group also received preoperative antibiotics (2 g cefazolin). Ten (76.9%) patients received indomethacin perioperatively (100 mg 48 h before and 50 mg 8 h after the procedure). No statistically significant correlation between perioperative antibiotics or indomethacin and PTB rate reduction was found (*p* > 0.05).

Twenty patients (28.6%) were assigned to a pessary placement. In two (2.9%) patients, the combination of the transvaginal cerclage and a pessary was used. In both cases, patients had experienced two previous second-trimester pregnancy losses, and the CL was 11 mm at 15 weeks and 17 mm at 16 weeks when the cerclage was placed. As the cervix continued to shorten, a pessary was placed at 20 weeks in both patients. The mean timing for a pessary placement was 21 weeks, and the average CL was 15 mm. In 12 (60.0%) patients in the pessary group, there were one or more PTB or second-trimester losses reported before.

We could not perform an independent comparison of the cerclage and pessary groups in terms of reducing the PTB as both groups also received progesterone. However, the progesterone therapy alone was associated with a significantly reduced PTB risk (x^2^(1) = 6.937, *p* = 0.008)) compared to the progesterone and cerclage or the progesterone and pessary.

### 3.4. The Pregnancy Outcomes

The pregnancy outcomes are shown in Table 3. In four cases PTB was iatrogenic: labour was induced between 32–36 + 6 weeks due to an intrauterine foetal death in one case; due to PPROM and oligohydramnios in another case; and gestational hypertension in another; in one patient, labour was induced at 26 + 2 weeks due to PPROM and oligohydramnios. The rates of PTB were 43.6% (*n* = 17), 45.5% (*n* = 5), 61.1% (*n* = 11), and 50.0% (*n* = 1) in the progesterone only, cerclage, pessary, and cerclage plus pessary groups, respectively. 

## 4. Discussion

Pregnancy, especially high-risk pregnancy, is not an unchanging constant but is a continuously fluctuating process depending on a number of etiological and external factors often playing in a stochastic manner. While several tools are available for maintaining pregnancies with a short cervix or CI, the lack of the optimal management strategy on how to maintain this high-risk pregnancy throughout the term makes this a clinically challenging task. The purpose of this study was to assess the key risk factors in the existing evaluation and management strategies as well as the pregnancy outcomes among patients with cervical shortening during pregnancy in a single centre in Latvia. This is the first work of its kind in our population on pregnant women with a particularly burdened clinical presentation.

### 4.1. Intra-Amniotic Infection/Inflammation as the Main Diagnostic and Therapeutic Challenge

It is well known that IA infection and sterile inflammation are important challenges among patients with cervical shortening, both for PTB risk assessment and management [4,22]. IA infection and sterile inflammation are one of the major causes of cervical shortening leading to PTB and are associated with a poor prognosis [4]. In fact, IA infection and sterile inflammation are the etiological factors underlying most deliveries before 32 weeks of pregnancy [23]. Based on previous studies, IA infection ascertained by amniocentesis has been reported in 52% of patients and IA inflammation in 81% of patients with cervical shortening [24,25]. In our study, PTB and second-trimester pregnancy loss were more common in the IA infection/inflammation group than in the non-infection/inflammation group, with the majority of PTB occurring before 28 weeks of gestation (70.4% vs. 41.9%; *p* = 0.048); in the group with PTB before 28 weeks, the IA infection/inflammation was observed in 10 patients (71.4%). As expected, the mortality rate was higher within the IA infection/inflammation group than in the non-infection/inflammation group (18.5% vs. 4.7%; *p* = 0.049). Five (71.4%) perinatal mortality cases and ten (58.8%) cases of 1 min Apgar score <7 were also observed in the IA infection/inflammation group. As PTB before 28 weeks is associated with the highest perinatal mortality and morbidity, patients in this group could benefit the most from an early and accurate diagnosis of IA infection/inflammation and antibiotic administration, with the aim of prolonging the pregnancy and improving the outcomes [23].

In our study, the diagnosis of IA infection/inflammation was based on the indirect signs—sludge in the amniotic fluid, cervical dilatation, and bulging membranes. The amniotic fluid sludge is detected by ultrasound and is associated with a microbial invasion into the amniotic cavity and histological chorioamnionitis and, thus, per se, reflects IA infection and inflammation [26]. IA infection and inflammation, in turn, lead to cervical shortening and bulging membranes [4,26]. Our results demonstrate that the most significant risk factors of PTB are cervical dilatation of ≥2 cm and bulging membranes (*p* = 0.01; φ = 0.3; OR 2.9 95%; CI [1.2–6.9] and *p* = 0.031; φ = 0.3; OR 3.3 95%; CI [1.0–10.7], respectively), bringing these two clinical manifestations to the forefront in the assessment of PTB risks.

Studies demonstrate that patients with cervical insufficiency, IA infection, and inflammation can be successfully treated with antimicrobial agents [23,24]. Recent clinical evidence indicates that the combined administration of ceftriaxone, clarithromycin, and metronidazole in a patient with a laboratory-confirmed IA inflammation or infection can resolve 75% of cases and prevent up to 40% of deliveries before 34 weeks [23]. In total, 65.7% of patients in our study received antibacterial therapy. Unfortunately, there was a lack of a unified approach to the choice of antibacterial therapy. In some cases, the decision for antibacterial therapy was at the discretion of the attending physician; as a result, some patients outside the IA infection/inflammation group also received antibacterial therapy. Nevertheless, our results demonstrate no reduction in the PTB rate in any of these groups receiving antibacterial therapy. In any case, we anticipate that the diagnosis of IA infection and sterile inflammation will be based on a more precise diagnostic approach, so antibacterial therapy could be reserved for cases with strict indications, thus reducing an unjustified antibacterial therapy and its associated complications. Currently, the gold standard to confirm IA infection/inflammation is based on objective laboratory evidence of infection/inflammation markers in the amniotic fluid obtained by an amniocentesis [10,27]. The implementation of amniocentesis as a diagnostic tool into routine clinical practice could facilitate an indication-based antibacterial therapy, also tailor making personalised treatment specifically to the causative microorganisms possible [27]. However, amniocentesis constitutes an invasive technique with known associated risks; therefore, the procedure has not received widespread acceptance in all patients, and less invasive and more rapid tests to detect an infection or inflammatory process are required [27].

### 4.2. Progesterone as the First Line of Treatment

As already described, the toolkit for maintaining a pregnancy with a short cervix or CI is limited to drug therapy (progesterone and antimicrobial agents), as well as mechanical prevention (pessary and cerclage). However, there are difficulties in identifying the population who would benefit the most from each intervention [28].

It was shown that cerclage in patients with a previous spontaneous PTB and cervical shortening reduces the rate of PTB before 35 weeks by 30% [17,28]. There is a lack of consensus on the optimal technique and the timing of the placement, as well as the role of amniocentesis before the cerclage’s placement, indomethacin, and antibacterial therapy and optimal care following the placement of a cerclage [29,30]. In our study, the mean gestational age for the cerclage placement was 17 weeks (with an average CL of 15 mm). Taking into consideration the results from recent studies, a cerclage remains effective in reducing PTB among patients with CL < 15 mm [29]. Cerclage has been also associated with superior neonatal outcomes compared to progesterone in patients with CL < 8–10 mm [29]. 

As it is highly likely that many patients with CL < 5 mm have an IA infection and/or inflammation, they may have been ineligible for a cerclage placement; in turn, this is a group of patients that might benefit from amniocentesis before placement of a cerclage to exclude an IA infection or inflammation [4,22]. Patients with cervical shortening without an IA infection or inflammation who undergo a cerclage placement have better pregnancy outcomes [22]. Therefore, it is pivotal to exclude IA infection or inflammation before the placement of a cerclage [22]. In our study, complications were observed in two patients (2/13) in the cerclage group (endometritis in both cases and sepsis in one case). In any case, the decisions about cerclage placement should be reserved only for cases with strict indications.

The use of a pessary has been proposed as an effective, inexpensive, and easy-to-implement method for prolonging pregnancy in patients with cervical shortening [31,32]. Its efficacy is not supported by the meta-analyses of randomised trials, although some individual trials have reported a reduction in the PTB rates at <34 weeks [6,33]. In our study, the mean gestational age for a pessary placement was 21 weeks, and on average, the CL was 15 mm. Overall, the evidence regarding the use of a pessary in patients with cervical shortening appears contradictory and should be revised [29]. In our study, a pessary or a cerclage was not associated with better pregnancy outcomes (despite also being on progesterone), mainly because these patients initially presented the poorest clinical indications. 

Numerous studies demonstrate that vaginal progesterone for patients with cervical shortening in the second trimester is associated with a significant reduction in the risk of PTB before 33 weeks, respiratory distress syndrome, intraventricular haemorrhage, necrotizing enterocolitis, neonatal sepsis, and birthweight of <1500 g and <2500 g, as well as admission to the neonatal intensive care unit [15,34,35]. Furthermore, there are no demonstrable deleterious effects on childhood neurodevelopment or maternal health in patients with progesterone therapy [36]. Consequently, all 70 (100%) patients in our study received progesterone supplementation, eventually leading to the best pregnancy outcomes and confirming that progesterone should remain the first line of treatment.

### 4.3. The Current Situation in the World Is Changing

The median PTB rate in Europe in 2015 was 7.3%, ranging from less than 6% in Latvia, Estonia, Finland, Sweden, and Lithuania to more than 8% in Belgium, Scotland, Romania, Germany, Hungary, Greece, and Cyprus [2]. In 2020, at the Riga Maternity hospital (which is the largest perinatal centre in our country), there were 5312 births with a PTB rate of 6.2%, reflecting one of the lowest PTB rates across Europe [1,2], although the PTB rate across the current study population was incomparably higher (48.6%) due to the fact that we focused our study on a particularly high PTB risk patient group with aggravated anamnesis (47.1% of our patients had at least one previous PTB or second-trimester pregnancy loss). The number of non-clinical trial studies focusing on real-life clinical scenarios is limited; therefore, obtaining a real comparable number of PTB rates is easier said than done. (Clinical trials primarily present their data in terms of the relative risk of PTB reduction.) The available numbers of PTB among patients treated with progesterone are 44.6, 34.8, and 27% [37,38,39]. It is noted that, in general, PTB rates in patients with a short cervix are high despite progesterone therapy.

Overall, our study results draw a heterogeneous picture of the real-world management of patients with cervical shortening. This might be partially due to the fact that, until recently, there has been no formal consensus on the ideal interventions for the management of women with cervical shortening during pregnancy. Fortunately, at the time of writing this article, the ISUOG has published Practice Guidelines providing healthcare practitioners with a consensus-based approach for the screening, follow-up, and treatment of patients at risk of PTB, including the ones with a short cervix and cervical insufficiency [17]. We are looking forward to implementing the uniform and evidence-based treatment strategy in Latvia as well, with the hope that this will help in prolonging high-risk pregnancies till term, ultimately reducing the costs of treatment and the rehabilitation of children born preterm and, most importantly, alleviating the suffering of patients. Furthermore, our study confirmed that there is often a multitude of individual risk factors for PTB, and emphasis should be placed on personalising medical therapy rather than on treating all high-risk women in the same manner.

## 5. Conclusions

A short cervix and bulging membranes, both indicators of intra-amniotic infection/inflammation, are the key risk factors in PTB risk prediction. Progesterone supplementation should remain at the forefront of PTB prevention. Among patients with a short cervix, especially complex anamnesis, the PTB rates remain high. The successful management of patients with cervical shortening lies between a consensus-based approach for screening, follow-up, and treatment on the one side and personalising medical therapy on the other.

## Figures and Tables

**Table 1:** The baseline characteristics of the study population (*n* = 70).

Variables	Results
Mean maternal age (years ± SD)	33.2 ± 5.43
Mean body mass index (kg/m² ± SD)	25.7 ± 5.7
Primigravida (*n* (%))	14 (20)
Multigravida (*n* (%))	56 (80)
No previous PTB or second-trimester pregnancy loss (*n* (%))	23 (41)
1 previous PTB or second-trimester pregnancy loss (*n* (%))	27 (48)
>1 previous PTB or second-trimester pregnancy loss (*n* (%))	6 (11)
Gestational weeks at inclusion (weeks ± SD)	21.6 ± 3.45
Cervical length at inclusion (mm ± SD)	12.5 ± 7.6
Obstetric history-based diagnosis (*n* (%))	5 (7.1)
Ultrasound-based diagnosis (*n* (%))	54 (77.1)
Physical examination-based diagnosis (*n* (%))	11 (15.8)
Risk factors of structural cervical weakness (a dilation and curettage, a hysteroscopy, a loop electrosurgical excision procedure, or a conization, or a congenital uterine anomaly) (*n* (%))	56 (80)
Complaints (pelvic pressure, weak irregular contractions, premenstrual-like cramping, and/or backache, and/or change in vaginal discharge) at inclusion (*n* (%))	22 (31.4)

PTB—preterm birth; SD—standard deviation.

**Table 2:** The comparison of the intra-amniotic infection/inflammation and the non-infection/inflammation group.

Variables	Intra-amniotic Infection/ Inflammation Group (*n* (%))	Non-Infection/Inflammation Group (*n* (%))
Complaints (pelvic pressure, weak irregular contractions, premenstrual-like cramping, and/or backache, and/or change in vaginal discharge) at the time diagnosis was made	6 (22.2)	16 (37.2)
Preterm birth or second-trimester pregnancy loss	19 (70.4)	18 (41.9) *
Preterm birth		
22 + 0–27 + 6 weeks	10 (37.1)	4 (9.3) *
28 + 0–31 + 6 weeks	3 (11.1)	3 (7.0) *
32 + 0–36 + 6 weeks	3 (11.1)	11 (25.6) *
Second-trimester loss	3 (11.1)	0 *
Term birth	8 (29.6)	25 (58.1) *
1 min Apgar score <7	10 (37.0)	7 (16.3)
Perinatal mortality	5 (18.5)	2 (4.7) *
Maternal septic complications (chorioamnionitis, endometritis, sepsis)	3 (11.)	3 (7.0)

* Statistically significant difference between groups.

**Table 3:** The pregnancy outcomes in the study population.

Variables	Results
Term birth (*n*, %)	33 (47.1%)
Preterm birth (*n*, %)	34 (48.6%)
22 + 0–27 + 6 weeks	14 (20.0%)
28 + 0–31 + 6 weeks	6 (8.6%)
32 + 0–36 + 6 weeks	14 (20.0%)
Second-trimester pregnancy loss (*n*, %)	3 (4.3%)
1 min Apgar score <7 (*n*, %)	17 (24.3%)
Perinatal mortality (*n*, %)	7 (10.0%)

## Data Availability

All data were incorporated into the article.
